# Prevalence of *Helicobacter pylori *infection among new outpatients with dyspepsia in Kuwait

**DOI:** 10.1186/1471-230X-10-14

**Published:** 2010-02-03

**Authors:** Waleed M Alazmi, Iqbal Siddique, Nabeel Alateeqi, Basil Al-Nakib

**Affiliations:** 1 Thunayan Alghanim Center of Gastroenterology, Amiri Hospital, Kuwait city, Kuwait

## Abstract

**Background:**

Testing and treatment for *Helicobacter pylori *has become widely accepted as the approach of choice for patients with chronic dyspepsia but no alarming features. We evaluated *H. pylori *status among outpatients with uninvestigated dyspepsia in Kuwait.

**Methods:**

A prospectively collected database for 1035 patients who had undergone ^13^C-urea breath tests (UBT) for various indications was reviewed for the period from October 2007 to July 2009. The status of *H. pylori *in dyspeptic patients was determined by UBT.

**Results:**

Among the 362 patients who had undergone UBT for uninvestigated dyspepsia, 49.7% were positive for *H. pylori *(95% CI = 44%-55%) and the percentage increased with age (35.8% at 20-29 years, 95% CI = 25.4% - 47.2%; 59.3% at 30-39 years, 95% CI = 48.5% - 69.5%) (P = 0.013). The prevalence of *H. pylori *was 42.6% among Kuwaitis (95% CI = 35%-50%) and 57.6% (95% CI = 49.8%-65%) among expatriates (p = 0.004). The prevalence among males was 51.3%, while in females it was 48.6%.

**Conclusions:**

Almost half of the patients with dyspeptic symptoms in Kuwait were positive for *H. pylori*, though the prevalence varied with age and was higher among expatriates. The American Gastroenterology Association guidelines recommending testing and treatment for *H. pylori *for patients with uninvestigated dyspepsia should be endorsed in Kuwait.

## Background

*Helicobacter pylori (H. pylori) *is causally related to serious disorders of the upper gastrointestinal tract in adults and children. Over 50% of the world's population is infected, with the highest prevalence in developing countries [1]. Although some reports have shown that *H. pylori*-positive patients tend to have dyspepsia [2], the relationship between *H. pylori *and dyspepsia remains controversial. The 2005 American College of Gastroenterology (ACG) guidelines for the management of dyspepsia recommend testing for *H. pylori *infection among dyspeptic patients without alarming features as the preferred, most cost-effective approach [3].

It is important to recognize the high prevalence of *H. pylori *among dyspeptic patients in Kuwait from the standpoint of eradication cost. In 1998, 88.5% of patients in Kuwait with dyspeptic symptoms who were referred for endoscopy proved *H. pylori*-positive [4]. Accordingly, the testing and treatment strategy recommended by the ACG would be the most cost-effective approach to patients with uninvestigated dyspepsia. Proper guidelines for dyspepsia can only be established when the prevalence of *H. pylori *among dyspeptic patients is clarified. The present study evaluated *H. pylori *status among outpatients with dyspepsia at a tertiary referral center in Kuwait.

## Methods

### Subjects

We performed a retrospective analysis on the ^13^C-urea breath test (^13^C-UBT) database that had been prospectively collected for the period from October 2007 to July 2009. The database included each patient's age, sex, weight, previous treatment for *H. pylori*, previous endoscopy, indication for UBT, and the ^13^C-UBT result. The Ethics Committee of the Kuwait Ministry of Health approved this study.

### Evaluation of *H. pylori *status with ^13^C-UBT

Patients aged 9 years or over ingested 100 mg of ^13^C-urea (Isomed, Madrid, Spain) in 75 ml of water after an 8-hour fast, then rinsed their mouths three times with tap water to minimize interference from oral urease-producing bacteria. Breath samples were collected into 250-ml siliconized vacutainers at baseline and at 30 min after the intake of ^13^C-urea. The ^13^CO_2_/^12^CO_2 _ratio was measured using an isotope ratio mass spectrometer (ABCA-G; Europa Scientific, Cheshire, UK). The increase in the molar fraction of tracer ^13^CO_2 _at 30 min compared with the baseline value was expressed as delta per ml (‰). In this study we took 3.5‰ as the cutoff value.

### Statistics

We performed all statistical analyses using the *SPSS *statistical package for Windows; the 95% confidence interval for key proportions was calculated using the exact binomial distribution. The chi square test was used to test the differences in proportion when appropriate; differences with *P *< 0.05 were deemed significant.

## Results

**Figure 1:**
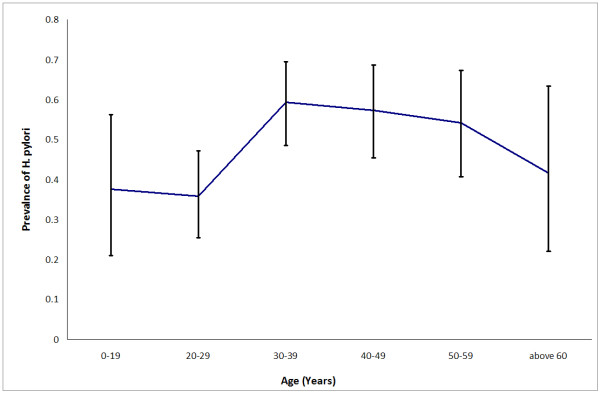
**Prevalence of *H. pylori *in 362 dyspeptic patients**.

The prospectively collected database for 1035 patients who had undergone a ^13^C-urea breath test for various indications was reviewed for the period from October 2007 to July 2009. In 362 of these 1035 patients (186 males and 176 females, mean age 38 years, range 10-80 years), UBT had been performed for uninvestigated dyspepsia. These patients were classed by nationality as Kuwaitis and expatriates, and according to age into young (less than 30 years), middle-aged (from 30 to 49 years) and elderly (more than 50 years). The overall prevalence of *H. pylori *among the 362 dyspeptic patients was 49.7% (95%CI: 44%- 55%). Figure (1) shows the variation of prevalence with age. The prevalence was 42.6% (95%CI: 35.5%- 50.0%) among Kuwaitis and 57.6% (95%CI: 49.8%- 65.0%) among expatriates (p = 0.004).

Among the dyspeptic Kuwaiti patients, *H. pylori *was significantly more prevalent among young females than males (38% Vs 10.5%, *p *= 0.008). However, there was no sex difference in overall prevalence (females 43.2%, males 42.2%) (Table 1). Among the dyspeptic expatriate patients, the overall prevalence of *H. pylori *was lower among females (40% vs 66%, *p *= 0.002) (Table 2).

**Table 1:** *H. pylori *prevalence in dyspeptic Kuwaiti patients by age and gender.

Age group	Female	Male	Total	*P *value
Young (<30 years)	38% (16/42)	10.5% (2/19)	29.5%	**0.008**
Middle aged (30-49 years)	50% (24/48)	48.5% (16/33)	48.2%	0.92
Elderly (>50 years)	39% (11/28)	63% (12/19)	40.6%	0.18
All	43.2% (51/118)	42.2% (30/78)		0.99

**Table 2:** *H. pylori *prevalence in dyspeptic non-Kuwaiti (expatriate) patients by age and gender.

Age group	Female	Male	Total	*P *value
Young (<30 years)	33% (8/24)	53.6% (15/28)	25%	0.22
Middle aged (30-49 years)	38% (8/21)	76.5% (49/64)	62%	**0.003**
Elderly (>50 years)	46% (6/13)	56.5% (12/23)	51.4%	0.795
All	40%(22/58)	66%(76/115)		**0.002**

Thirty-three patients (9%) infected with *H. pylori *needed upper endoscopy compared to 42 (6.2%) who were not infected. There were no statistically significant differences with age in the rate of endoscopy.

## Discussion

Dyspepsia remains a costly, chronic condition, and drug costs in particular continue to increase rapidly [5]. In many cases, the symptoms are of short duration or mild in severity and are self-managed [6]. The optimal management strategy for patients aged 55 years or younger who present with new-onset dyspepsia and no alarming features has been dominated by testing for *H. pylori *and treating all positive cases empirically with antimicrobial therapy. However, other strategies have been practiced such as empirical medical therapy (e.g. antisecretory agents), subsequent investigation being limited to failures, or immediate evaluation by upper endoscopy in all cases, therapy being targeted on the basis of the results [7].

The *H. pylori *testing and treatment approach is likely to be more beneficial than other strategies in infected patients, and the impact of this strategy is likely to be small if the infection is not very prevalent [7]. In addition, cost-effectiveness studies suggest that a choice of noninvasive testing should be based on the prevalence of infection in the community. In low and intermediate prevalence situations, the stool antigen test or urea breath test dominate [8]. The higher costs of these tests are offset by their accuracy. Accordingly, it is clear that determining the prevalence of *H. pylori *infection in the community is fundamental to deciding the most cost-effective strategy for managing patients with uninvestigated dyspepsia.

In 1998, 88.5% of patients in Kuwait with dyspeptic symptoms who were referred for endoscopy proved *H. pylori*-positive [4]. However, the prevalence of *H. pylori *infection has continued to decline dramatically, as has the identification of peptic ulcer disease [9]. Moreover, the prevalence of *H. pylori *infection differs widely among countries and by age and race [10]. Hence, our present study was designed to evaluate the prevalence of *H. pylori *infection among new dyspepsia patients and to stratify them by age and race in order to apply the appropriate management strategy.

The overall prevalence of *H. pylori *among the 362 dyspeptic patients in our study was 49.7%, though it varied with age, race and sex. This prevalence is considered intermediate. On the basis of this study, therefore, the choice of noninvasive test in our community should be either the urea breath test or stool antigen test, both of which have been shown to be accurate for the initial diagnosis of *H. pylori *infection and for confirming eradication [7]. The urea breath test is widely available in Kuwait in both governmental and private sectors, but the stool antigen test is not yet available. Both tests require discontinuation of PPI for two weeks because the drugs used inhibit urease, leading to false negative results [11].

In view of the current prevalence of *H. pylori *infection in Kuwait, the testing and treatment approach is likely to be the most cost-effective strategy for evaluating patients with dyspepsia. This policy would decrease the work-load on endoscopy centers, lower the costs and decrease the rate of complications associated with upper endoscopy. Moreover, the management approach can easily be established at primary care centers and general hospital clinics. This would decrease the number of patients in subspecialty clinics, allowing more time for the specialists to manage more complex gastrointestinal diseases.

Our study did not evaluate the relief of symptoms or healing rates in the dyspepsia patients after *H. pylori *infection was treated. Consequently, it did not confirm the usefulness of the testing and treatment strategy in clinical practice for relief of symptoms. Furthermore, in patients older than 50 years, direct endoscopy could be an alternative strategy for detecting gastroduodenal pathology, which may require close follow-up in this patient age group.

After stratifying the patients according to race, age and sex, it was evident that there was significantly more *H. pylori *infection among the expatriates (57.6% vs 42.6%, *p *= 0.004). Since most of the expatriates belong to either low or intermediate socioeconomic classes, a cost-effective approach to the management of dyspepsia would benefit this category of patients. Our data also indicated that among dyspeptic expatriates, *H. pylori *was less prevalent overall among female patients (40% vs 66%, *p *= 0.002). This sex difference in prevalence may be attributed to numerous epidemiological factors including country of origin, socioeconomic class, place of birth and ethnicity.

Among the dyspeptic Kuwaiti patients, on the other hand, *H. pylori *was significantly more prevalent among young females than males (38% vs 10.5%, *p *= 0.008), but there was no sex difference overall (females 43.2% Vs males 42.2%) (Table 1). This indicates that female Kuwaiti patients acquire *H. pylori *infection at a younger age than males, which could be translated clinically to a more aggressive disease associated with long duration of infection.

In a population-based study from Norway [12], the distribution of *H. pylori *infection with regard to dyspepsia in men and women was uneven. Moreover, the prevalence of *H. pylori *infection had decreased independently of dyspepsia, especially in younger age groups. These findings are broadly similar to those of our present study, questioning our understanding of the causal relationship between dyspepsia and *H. pylori *infection.

## Conclusions

The present study establishes the prevalence of *H. pylori *infection in dyspeptic patients in Kuwait. It also describes the variation in prevalence with age, sex and country of origin. Our findings support the American Gastroenterology Association guidelines, recommending testing and treatment for *H. pylori *infection in patients with new-onset dyspepsia as the most cost-effective approach.

## Competing interests

The authors declare that they have no competing interests.

## Authors' contributions

**WA **conceived the study, participated in its design and coordination and the acquisition of data, performed statistical analysis, and drafted the manuscript and critically revised it for important intellectual content. **IS **participated in the study design and statistical analysis. **NA **participated in acquisition of data and manuscript writing. **BA **participated in study design and coordination and acquisition of data, and critical revision of manuscript for important intellectual content.

All authors have read and approved the final manuscript

## Pre-publication history

The pre-publication history for this paper can be accessed here:

http://www.biomedcentral.com/1471-230X/10/14/prepub
